# Stabilization of Myc through Heterotypic Poly-Ubiquitination by mLANA Is Critical for γ-Herpesvirus Lymphoproliferation

**DOI:** 10.1371/journal.ppat.1003554

**Published:** 2013-08-08

**Authors:** Lénia Rodrigues, Nikita Popov, Kenneth M. Kaye, J. Pedro Simas

**Affiliations:** 1 Instituto de Medicina Molecular, Faculdade de Medicina, Universidade de Lisboa, Lisboa, Portugal; 2 Theodor Boveri Institute, Biocenter, University of Würzburg, Würzburg, Germany; 3 Channing Laboratory and Departments of Medicine, Brigham and Women's Hospital and Harvard Medical School, Boston, Massachusetts, United States of America; University of North Carolina, United States of America

## Abstract

Host colonization by lymphotropic γ-herpesviruses depends critically on expansion of viral genomes in germinal center (GC) B-cells. Myc is essential for the formation and maintenance of GCs. Yet, the role of Myc in the pathogenesis of γ-herpesviruses is still largely unknown. In this study, Myc was shown to be essential for the lymphotropic γ-herpesvirus MuHV-4 biology as infected cells exhibited increased expression of Myc signature genes and the virus was unable to expand in Myc defficient GC B-cells. We describe a novel strategy of a viral protein activating Myc through increased protein stability resulting in increased progression through the cell cycle. This is acomplished by modulating a physiological post-translational regulatory pathway of Myc. The molecular mechanism involves Myc heterotypic poly-ubiquitination mediated via the viral E3 ubiquitin-ligase mLANA protein. EC_5_S^mLANA^ modulates cellular control of Myc turnover by antagonizing SCF^Fbw7^ mediated proteasomal degradation of Myc, mimicking SCF^β-TrCP^. The findings here reported reveal that modulation of Myc is essential for γ-herpesvirus persistent infection, establishing a link between virus induced lymphoproliferation and disease.

## Author Summary

Being obligatory intracellular parasites, it is not surprising that viruses have evolved mechanisms to induce cellular proliferation to promote their own life cycle. This is notorious in the case of γ-herpesviruses, such as Epstein-Barr virus (EBV) and Kaposi's sarcoma virus (KSHV), which are human pathogens associated with lymphoproliferative disease and several tumors. Host colonization by γ-herpesviruses is critically dependent on the ability to expand latent infection in proliferating B-cells. Virus-induced cellular proliferation is a process mediated by the expression of specific viral proteins. One of such proteins is the latency-associated protein (LANA) of KSHV. In this study, we use murid herpesvirus-4 (MuHV-4) as a mouse model of γ-herpesvirus pathogenesis. We show that the MuHV-4 LANA (mLANA) stabilizes the cellular oncogene Myc, increasing its half-life, thus promoting its activity as a potent inducer of cellular proliferation. The molecular mechanism involves heterotypic poly-ubiquitination of Myc mediated via mLANA. The findings here reported demonstrate that modulation of Myc is essential for γ-herpesvirus persistent infection, establishing a link between virus induced lymphoproliferation and disease. The implication is that revealing a critical function of a viral protein possibly allows the development of small molecule probes to disrupt mLANA-Myc interaction, therefore inhibit virus induced lyhophoproliferative disease.

## Introduction

Myc is a transcription factor that enhances the expression of genes involved in cellular growth and proliferation. Hence, it is not surprising that viruses have evolved mechanisms to modulate Myc to promote their own life cycle. Myc heterodimerizes with Max, through a basic region/helix-loop-helix/leucine-zipper domain, to regulate the transcription of specific E-box-containing genes in response to mitogenic stimuli. Myc functions as a universal amplifier of gene expression by promoting the transcriptional elongation of RNA polymerase II driving biomass accumulation and enhanced cellular bioenergetic pathways [1], [2], [3]. The expression of *c-myc* is tightly regulated with extremely short half-lives for mRNA and protein. In non-transformed cells, Myc is continuously subjected to ubiquitination and proteasomal-degradation, resulting in a highly unstable protein with a half-life of about 15–20 minutes [4]. Several mechanisms of Myc regulation have been identified that operate at the level of protein stability. The best characterized mechanism involves the interplay between phosphorylation at two specific residues and ubiquitination. Phosphorylation at serine (S) 62 by extracellular signal-regulated kinase (ERK) stabilizes Myc resulting in enhancement of its transcription activity. In contrast, phosphorylation of Myc at threonine (T) 58 by glycogen synthase kinase 3 (Gsk-3), which is dependent on previous phosphorylation of Myc at S62, leads to proteasomal degradation of Myc [5]. The mechanism involves the assembly of homotypic poly-ubiquitin chains on Myc specifically dependent on lysine (K) 48 linkage by SCF (Skp1/Cul/Fbox)^Fbw7^ [6], [7]. Myc turnover by SCF^Fbw7^ is antagonized by polymerization of mixed heterotypic poly-ubiquitination chains via SCF^β-TrCP^ on the N-terminus of Myc [8]. Thus, SCF^Fbw7^ and SCF^β-TrCP^ assemble different K-linkage poly-ubiquitin chains with functionally distinct outcomes on Myc stability, i.e., degradation versus stability. The physiological relevance of regulating Myc activity through protein stability is underscored by observations that point mutations at or near T58, which render Myc resistant to proteasomal degradation, occur with high frequency in B-cell lymphomas [9].

Examples of viruses that modulate Myc activity include Kaposi's sarcoma associated herpesvirus (KSHV) and Epstein-Barr virus (EBV). Infection by these γ-herpesviruses is characterized by the establishment of latent infection in memory B-cells. Access to this cell type is gained by virus-driven proliferation of germinal centre (GC) B-cells [10], where virus genomes replicate and segregate in step with normal cell division. This process is mediated by episomal maintenance proteins, which include EBV nuclear antigen-1 (EBNA-1) [11] and latency associated nuclear antigen (LANA) encoded by *ORF73* of γ-2-herpesviruses [12]. Given the essential role of Myc for the initiation and maintenance of GCs [13], [14], it is not surprising that γ-herpesviruses have evolved mechanisms to modulate Myc activity. In the case of KSHV-associated primary effusion lymphoma, Myc was shown to be abnormally stabilized [15], [16]. The mechanism involves the direct interaction of the viral protein LANA with Gsk-3 resulting on reduced levels of Myc T58 phosphorylation [17]. Another strategy appears to be employed by EBV encoded EBNA-3C protein. This viral protein was shown to increase the transcriptional activity of Myc through an interaction with both Myc and SCF^Skp2^. Surprisingly the mechanism proposed does not involve poly-ubiquitination but rather the action of SCF^Skp2^ functioning as a transcription co-factor for Myc [18].

Here, we utilized murid herpesvirus-4 (HuHV-4) infection of mice as a model system to address the role of Myc in the pathogenesis of γ-herpesviruses. We show that Myc expression is required for the expansion of MuHV-4 infection in GC B-cells. The mechanism involves heterotypic poly-ubiquitination of Myc mediated through the ElonginC/Cullin5/SOCS (supressors of cytokine signaling) (EC_5_S) E3 ubiquitin-ligase activity of mLANA encoded by *ORF73* of MuHV-4. EC_5_S^mLANA^ mimics SCF^β-TrCP^ by antagonizing SCF^Fbw7^-mediated proteasomal turnover of Myc but unlike the cellular E3 ubiquitin-ligases its activity is not dependent on the phosphorylation status of Myc. Our results underscore the importance of modulating Myc activity during γ-herpesvirus driven lymphoproliferation providing a link between persistent infection and lymphoproliferative disease.

## Results

### Myc transcriptional activity is up-regulated during MuHV-4 infection of GC B-cells

**Figure 1:**
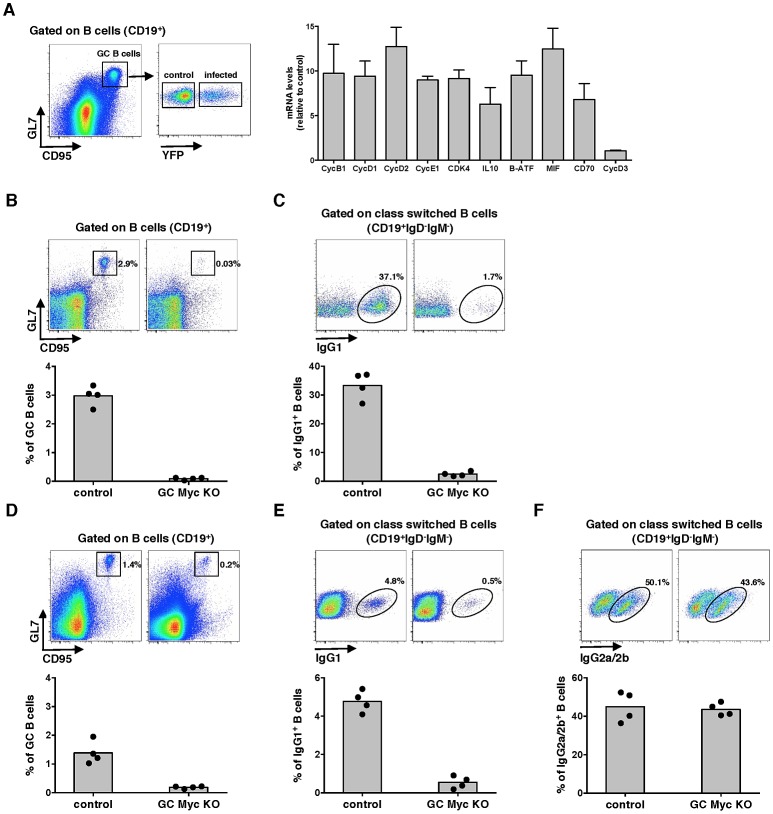
Myc transcriptional activity is upregulated during MuHV-4 latency in GC cells. (A) MuHV-4 infected cells exhibit augmented expression of Myc-dependent genes. FACS purified infected (CD19^+^CD95^hi^GL7^hi^YFP^+^) or control uninfected (CD19^+^CD95^hi^GL7^hi^YFP^−^) GC B-cells from C57BL/6 mice infected with MuHV-4-YFP were analysed at 13 days pi for transcription of genes encoding for cyclins (Cyc) B1, D1, D2, D3, E1, cyclin-dependent kinase (Cdk) 4, IL10, B-ATF, MIF and CD70 by qPCR. Transcription of each gene, normalized to GAPDH, is represented as fold induction relative to control uninfected cells purified form the same pool of 5 spleens. Error bars represent SEM from three independent experiments. (B and C) *Cγ1-cre^KI/WT^*; *c-myc^fl/fl^* (GC Myc KO) mice are unable to mount a GC reaction. GC Myc KO and control mice immunized with NP-CGG were analysed at day 10 post-immunization for frequencies of GC (CD19^+^CD95^hi^GL7^hi^) and IgG1 (CD19^+^IgD^−^IgM^−^IgG1^+^) B-cells, by flow cytometry. (D, E and F) GC Myc KO mice exhibit normal numbers of IgG2a/2b B-cells. GC Myc KO and control mice immunized with CFA-OVA were analysed 14 days later for frequencies of GC, IgG1 and IgG2a/2b (CD19^+^IgD^−^IgM^−^IgG2a/2b^+^) B-cells by flow cytometry. Each point represents an individual mouse; four mice (n = 4) were analyzed for each experimental condition; grey bars indicate the mean. Representative FACS plots from individual animals are shown (top panels).

Experiments were designed to investigate the role of Myc expression on gammaherpesvirus pathogenesis. We utilized MuHV-4 infection of laboratory mice as the model, which is characterized by the expansion of latently infected B-cells in GCs and virus persistence in memory B-cells [19]. We analysed the transcription of several Myc target genes in infected versus non-infected GC B-cells, purified from the same pool of splenocytes. We utilized a recombinant MuHV-4 expressing a yellow fluorescent protein (YFP) [20] to segregate infected (CD19^+^CD95^hi^GL7^hi^YFP^+^) from non-infected (CD19^+^CD95^hi^GL7^hi^YFP^−^) GC B-cells derived from C57BL/6 mice, at day 13 post-infection. We analysed the transcription of Myc signature genes involved in cell cycle entry (encoding for cyclins B1, D1, D2 and E, and cyclin-dependent kinase 4) and GC B-cell activation (encoding for IL-10, B-ATF, MIF and CD70). Cyclin D3 was included as a gene non-regulated by Myc. The transcription of Myc target genes was significantly increased in infected GC B-cells when compared with their non-infected counterparts (Figure 1A). These data show that during the expansion of latent infection in GC B-cells, MuHV-4 induces a transcription profile compatible with increased Myc transcriptional activity.

### Myc is essential for GC responses

To define the impact of Myc expression on gamma-herpesvirus pathogenesis, we generated mice with conditional deletion of *c-myc* in B-cells undergoing GC reaction. This was achieved by breeding homozygous *c-myc^fl/fl^* mice, where second and third exons of the *c-myc* locus are flanked by two loxP sites [21] to heterozygous *Cγ1-cre* mice, in which expression of Cre recombinase is induced by transcription of the Ig-γ1 constant region gene segment early in GC development during immunoglobulin class-switch recombination [22]. Resulting progeny *Cγ1-cre^KI/WT^*;*c-myc^fl/fl^*, hereafter designated GC Myc KO, is expected to have specific deletion of *c-myc* in class-switched GC B-cells, after immunization with T-dependent antigens. Thus, we next investigated GC responses in the absence of Myc expression. GC Myc KO mice were immunized with the Th-2 cell-dependent antigen 4-hydroxy-3-nitrophenylacetyl (NP)–chicken γ-globulin (CGG) adsorbed to alum. Frequencies of GC B-cells (CD19^+^CD95^hi^GL7^hi^) were analysed at day 10 post-immunization. Compared to control litter mates *Cγ1-cre^WT/WT^*;*c-myc^fl/fl^*, hereafter designated control mice, GC Myc KO mice showed a marked impairment in GC development (Figure 1B), accompanied by a strong reduction in IgG1^+^ B-cell numbers (CD19^+^IgD^−^IgM^−^IgG1^+^) (Figure 1C). Immunization with ovalbumin (OVA) emulsified in complete Freund's adjuvant (CFA) further confirmed that GC Myc KO mice were defective to mount a normal GC response, though less deficient than upon immunization with NP-CGG, and presented reduced levels of IgG1 expressing B-cells (Figure 1D and 1E, respectively). However frequencies of IgG2a/2b^+^ B-cells (CD19^+^IgD^−^IgM^−^IgG2a/2b^+^), whose class-switching is not strictly dependent on Ig-γ1 promoter, revealed that GC Myc KO mice are competent in developing IgG2 class-switched B-cells (Figure 1F). These data demonstrate a clear requirement for Myc expression in order to generate GC reactions to T cell dependent antigens. Our results are in direct agreement with those recently published that demonstrate the lack of GCs in mice in which *c-myc* is ablated early during GC induction [13]. Since we obtained the two transgenic mice lines carrying the alleles *c-myc^fl/fl^* and *Cγ1-cre* from these authors, and independently generated GC Myc KO mice, our data are directly comparable.

### Myc is critical for the establishment of persistent infection

**Figure 2:**
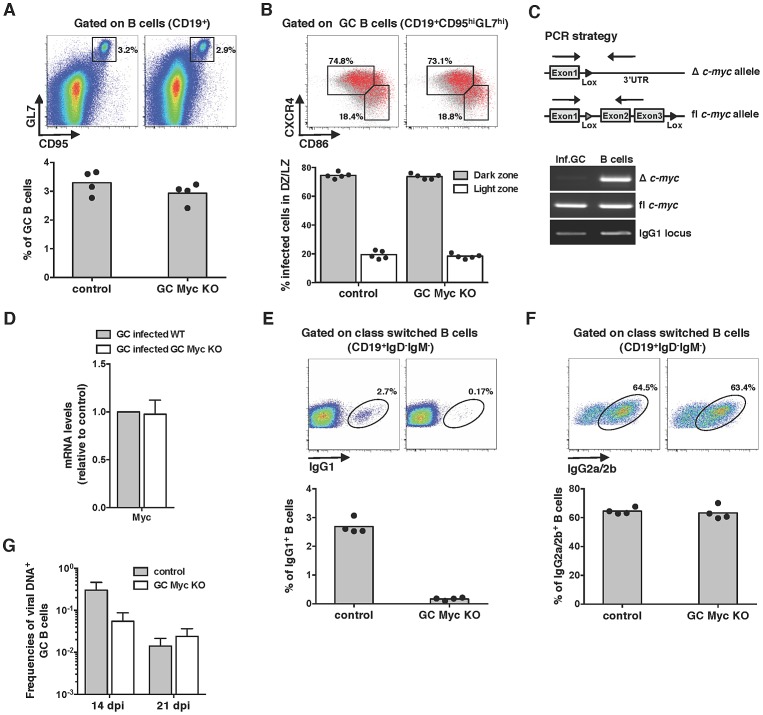
Myc is essential for the amplification of MuHV-4 infection in GC B-cells. (A and B) MuHV-4 infection of GC Myc KO mice leads to the development of normal GC reactions. (A) GC B-cell percentages were determined by flow cytometry as described in Figures 1B and 1D at day 14 pi. (B) Dark zone (CD19^+^CD95^hi^GL7^hi^CXCR4^hi^CD86^lo^) and light zone (CD19^+^CD95^hi^GL7^hi^CXCR4^lo^CD86^hi^) percentage of infected cells at day 14 pi. Each point represents an individual mouse; five mice (n = 5) were analyzed for each experimental condition. (C and D) MuHV-4 infected cells from GC Myc KO mice are genotypically and phenotypically wild type for Myc. (C) Total DNA from FACS purified MuHV-4 infected GC B-cells, from GC Myc KO mice, was subjected to PCR analysis with primers specific for deleted (Δ) or floxed (fl) *c-myc*, as depicted. DNA from total B-cells, in the same pool of splenocytes, was analysed in parallel as a positive control for Δ *c-myc*. (D) Transcription of Myc, normalized to GAPDH, in purified GC B-cells from GC Myc KO mice is represented as fold induction relative to MuHV-4 infected GC B-cells purified from wild type mice. Error bars represent SEM from three independent experiments. (E and F) MuHV-4 infection induces a strong IgG2a/2b response. GC Myc KO and control mice were analysed at day 21 pi for frequencies of IgG1 and IgG2a/2b B-cells by flow cytometry. Each point represents an individual mouse; four mice (n = 4) were analyzed for each experimental condition; grey bars indicate the mean. Representative FACS plots from individual animals are shown (top panels). (G) MuHV-4 infection of GC Myc KO mice reveals a deficit in the establishment of latency. GC Myc KO and control mice were analysed at days 14 and 21 pi for frequencies of viral infection in GC B-cells. Data were obtained from pools of 5 spleens. Error-bars represent the frequency of viral DNA-positive cells with 95% confidence intervals.

The requirement of Myc for gammaherpesvirus pathogenesis was next investigated by infecting GC Myc KO mice with MuHV-4. Analysis of the percentages of GC B-cells revealed no significant differences between control and GC Myc KO mice (Figure 2A), with the majority of infected GC B-cells falling into dark zone (Figure 2B) as previously described for infection of wild type mice [23]. To determine if MuHV-4 infected GC B-cells had been or not exposed to Cre-mediated *c-myc* deletion, we FACS purified infected cells from GC Myc KO mice, at day 14 post-infection. A PCR assay was employed to detect floxed and deleted *c-myc* alleles in DNA from infected GC B-cells, compared to DNA from uninfected total B-cells (CD19^+^YFP^−^), purified from the same pool of splenocytes. Infected cells were found to have undeleted (floxed) *c-myc* allele and no *c-myc* rearrangement could be detected (Figure 2C). This contrasted with total non-infected B-cell population where *c-myc* deletion could be readily detected (Figure 2C). To further confirm the integrity of the *c-myc* locus in infected GC B-cells in GC Myc KO mice, we quantified Myc mRNA levels. Comparison of GC B-cells from GC Myc KO with wild type infected mice revealed no significant differences in Myc transcription (Figure 2D). These data imply that MuHV-4 is expanding exclusively in GC B-cells where the *c-myc* locus did not undergo Cre-mediated deletion. Accordingly, GC Myc KO mice infected with MuHV-4 were unable to class-switch to IgG1, which is consistent with Myc deficiency in these cells (Figure 2E). However, these infected mice showed high numbers of IgG2a/2b positive B-cells, equivalent to infected control mice counterparts (Figure 2F). Quantification of the frequency of viral DNA positive cells in GCs, assessed by limiting dilution combined with real time PCR, showed an approximately 10-fold deficit of latent infection at 14 days post-infection in GC Myc KO mice (Figure 2G). This deficit was likely a reflection of infection of B-cells that did not result in the expansion in GC reactions due to Ig-γ1 Cre mediated *c-myc* deletion. However, at day 21 post-infection latent viral loads in control and conditional KO mice were equivalent (Figure 2G), indicating a recovery from the early deficit in expansion. Collectively data obtained with GC Myc KO mice demonstrated that infection with MuHV-4 does not compensate for Myc loss in GC B-cells and virus is found to amplify exclusively in *c-myc* intact cells. Therefore, Myc is essential for the expansion of latently infected GC B-cells, thus critical for the establishment of persistent infection.

### mLANA promotes Myc transcriptional activity and cell cycle progression as an EC_5_S ubiquitin-ligase

**Figure 3:**
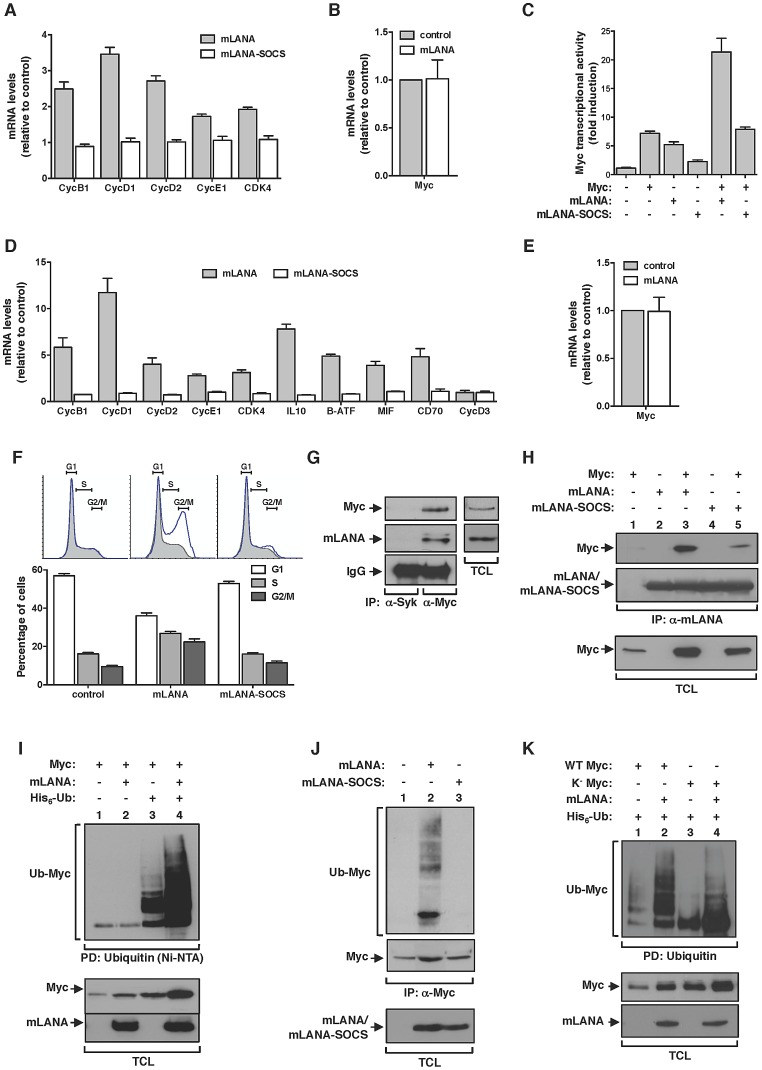
mLANA promotes Myc transcriptional activity and cell cycle progression through E3 ubiquitin-ligase activity. (A) mLANA expressing cells show increased expression of Cyclin (Cyc) and Cyclin-dependent kinase 4 (Cdk4) genes. HEK 293T cells were transfected as indicated and transcription of Myc target genes was analysed as described in Figure 1A. Error bars represent SEM from four independent transfection experiments. (B) mLANA expression does not affect Myc mRNA levels. Relative levels of Myc mRNA in mLANA-transfected are shown in comparison to control cells. Error bars represent SEM from four independent transfection experiments. (C) mLANA activates Myc transcriptional activity dependent on its SOCS-box motif. HEK 293T cells were transfected with a Myc (E-box) luciferase reporter vector and with the indicated expression plasmids. Error bars represent SEM from triplicates from three independent transfection experiments. (D) Expression of Myc signature genes involved in cell cycle entry and B-cell activation is increased under mLANA expression. A20 cells were transiently transfected with GFP-mLANA, GFP-mLANA-SOCS or control transfected. Transfected cells were FACS-purified based on GFP expression and proceeded to RNA extraction. Transcription of Myc target genes was analysed as described in Figure 1A. (E) mLANA expression in A20 cells does not affect Myc mRNA levels. Relative levels of Myc mRNA in mLANA expressing cells are shown in comparison to control cells. (F) Expression of mLANA promotes cell cycle progression through S and G2-M phases. Cell cycle profiles of A20 cells transfected with GFP, GFP-mLANA or GFP-mLANA-SOCS expressing plasmids were obtained by staining with Vybrant DyeCycle followed by FACS analysis. Shaded histograms show the cell cycle profile of GFP^−^ populations, whereas the blue lines represent GFP^+^ cells, for each transfection condition. The percentage of cells in each phase of cell cycle was determined using FlowJo software modelled by the Dean-Jett-Fox method. Three independent experiments were performed for each experimental condition, a representative of which is shown (top). Error bars represent SEM. (G and H) mLANA and Myc co-immunoprecipitate in the same heteromolecular complex. (G) Lysates from S11 cells persistently infected with MuHV-4 were immunoprecipitated with an anti-Myc and an irrelevant antibody (anti-Syk) as negative control and analyzed by immunoblotting as indicated. Representative aliquots of TCL were used to demonstrate expression of Myc and mLANA in this cell line. (H) HEK 293T cells transiently expressing the indicated combinations of proteins (top) were subjected to immunoprecipitation with anti-mLANA antibodies. The presence of co-immunoprecipitated Myc was assayed by immunoblotting with anti-Myc. (I) mLANA expressing cells present increased levels of Myc ubiquitination. HEK 293T cells were transiently transfected with the indicated plasmids (top). After culture, total cellular lysates were obtained and ubiquitinated proteins were pulled down using Ni-NTA beads. The levels of ubiquitinated Myc in each condition were assayed using an anti-Myc. (J) mLANA mediates ubiquitination of endogenous Myc. HEK 293T cells transiently expressing mLANA, mLANA-SOCS, or control cells were subjected to immunoprecipitation with an anti-Myc and analysed by immunoblotting with anti-ubiquitin (Ub). (K) mLANA is able to mediate the assembly of poly-ubiquitin chains on a lysine-free version of Myc (K^−^Myc). HeLa cells were transiently transfected to express the indicated combinations of plasmids (top). Ubiquitinated proteins were pulled-down using Ni-NTA beads and resolved by SDS-PAGE. Levels of ubiquitinated WT or K^−^ Myc were analysed by immunoblotting. −, without; +, with; α-, anti; IP, immunoprecipitation; PD, pull down; TCL, total cellular lysates.

We have shown before that the ORF73 protein encoded by MuHV-4, designated mLANA by homology with the latency associated nuclear antigen encoded by KSHV, is selectively transcribed in GC B-cells [24]. Thus mLANA was a strong candidate to be responsible for the observed increased transcription of Myc target genes in MuHV-4 infected GC B-cells. To address this hypothesis, we analysed the transcription of Myc target genes in mLANA expressing cells. When compared to control transfected cells, mLANA expression induced the transcription of all Myc target genes analysed (Figure 3A). Expression of mLANA had no effect on *c-myc* mRNA levels indicating that its putative modulatory effect on Myc was post-transcriptional (Figure 3B). We have also shown before that mLANA acts as the substrate recognition factor of an ElonginC-Cullin5-SOCS (suppressor of cytokine signalling) (EC_5_S) E3 ubiquitin-ligase towards the p65/RelA cellular transcription factor NF-κB [25]. The mechanism involves the assembly of an EC_5_S -like complex, mediated by a viral unconventional SOCS-box motif present in mLANA. Hence, we analysed if mLANA-mediated modulation of transcription of Myc target genes could be attributed to its function as an E3 ubiquitin-ligase. To this end, we utilized a previously characterized mLANA mutant, designated mLANA-SOCS where residues V199, L202, P203 and P206 were substituted by alanines abrogating E3 ubiquitin-ligase function [25]. This mutant was no longer able to modulate the expression of Myc target genes when compared with intact mLANA (Figure 3A). To define if the observed mLANA modulatory effect on cellular transcription was Myc specific, we carried out gene reporter assays using a synthetic Myc reporter plasmid containing three copies of E-box sequences driving the expression of luciferase. As expected, overexpression of Myc was translated into a significant increase on luciferase activity (Figure 3C). Cells expressing mLANA exhibited comparable levels of luciferase activity, which increased further when Myc was concomitantly expressed. In contrast, mLANA-SOCS expression showed no effect on luciferase levels. We next proceeded to analyse the modulatory effect of mLANA on Myc in B-cells, which are physiological more relevant given the tropism of MuHV-4. When compared to control and mLANA-SOCS transfected cells, mLANA expression induced the transcription of all Myc target genes analysed (Figure 3D). As before, expression of mLANA in A20 B-cells had no effect on *c-myc* mRNA levels confirming that its modulatory effect on Myc was post-transcriptional (Figure 3E). Myc transcriptional activation of genes encoding proteins involved in cell cycle entry results in transition from G0-G1 to S phase. Thus, we analysed cell cycle profiles in B-cells expressing mLANA in comparison to control or mLANA-SOCS. These experiments showed a clear decrease in the number of mLANA expressing cells in G1 phase, with a concomitant increase in the number of cells in S and G2-M phases (Figure 3F). Combined these data indicate that mLANA is modulating Myc-dependent transcription and progression through cell cycle in B-cells through its activity as an EC_5_S E3 ubiquitin-ligase. Co-immunoprecipitation experiments also showed that mLANA and Myc exist in the same heteromolecular complex in a context of virus infection. This was demonstrated using a murine B-cell lymphoma-derived cell line latently infected with MuHV-4, designated S11 cells (Figure 3G). We also showed that this interaction was reduced for mLANA-SOCS (Figure 3H, compare lanes 3 and 5). This reduction could be due to lower levels of Myc in mLANA-SOCS expressing cells, when compared to mLANA, indicating that the interaction is independent of the SOCS-box motif. Alternatively it is plausible that the SOCS-box is participating in mLANA-Myc interaction.

### mLANA mediates Myc poly-ubiquitination

We next set out experiments to investigate if EC_5_S^mLANA^ was able to mediate poly-ubiquitination of Myc. We started by performing a nickel-nitrilotriacetic acid (Ni-NTA) pull-down in the presence of histidine-tagged ubiquitin. Upon culture and cell lysis, ubiquitinated proteins were extracted from total cellular lysates with Ni-NTA beads and resolved by SDS-PAGE. The levels of ubiquitinated Myc present in each condition were analysed by immunoblotting. We observed that when mLANA was expressed, the levels of ubiquitinated Myc were significantly enhanced (Figure 3I, compare lanes 3 and 4). To confirm this activity in a more relevant biological context, we evaluated the ability of mLANA to promote the ubiquitination of endogenously expressed Myc. In comparison with control transfected and mLANA-SOCS transfected cells, higher levels of ubiquitinated Myc were detected in the presence of intact mLANA (Figure 3J, compare lane 2 with 1 and 3). We have previously demonstrated that mLANA E3 ubiquitin-ligase activity towards p65/RelA required the E2 ubiquitin-conjugating enzyme UbcH5 [25]. Here we showed that EC_5_S^mLANA^ requires UbcH5 as its E2 conjugating partner to mediate poly-ubiquitination of Myc (Figure S1). Myc protein has 25 lysine residues that can be potentially ubiquitinated. To define if the ubiquitination ladder observed was due to the ability of mLANA to mediate poly-ubiquitination or multiple mono-ubiquitination of Myc in different lysine residues, we made use of a previously described lysine-free (K^−^) version of Myc, which can only be ubiquitinated on its N-terminal residue [8]. By performing an in vivo ubiquitination assay with K^−^Myc, we observed the following. First, mLANA was able to mediate poly-ubiquitination of the N-terminal residue of Myc (Figure 3K, compare lanes 3 and 4). Secondly, K^−^Myc in the presence of mLANA exhibited a ladder of ubiquitination indicating the assembly of poly-ubiquitin chains (Figure 3K, compare lanes 3 and 4). Finally, the ubiquitination pattern of wild type Myc and K^−^Myc are distinctive, implicating that other lysine residue(s) are targets for mLANA-mediated poly-ubiquitination of Myc.

### EC_5_S^mLANA^ assembles heterotypic poly-ubiquitin chains on Myc

**Figure 4:**
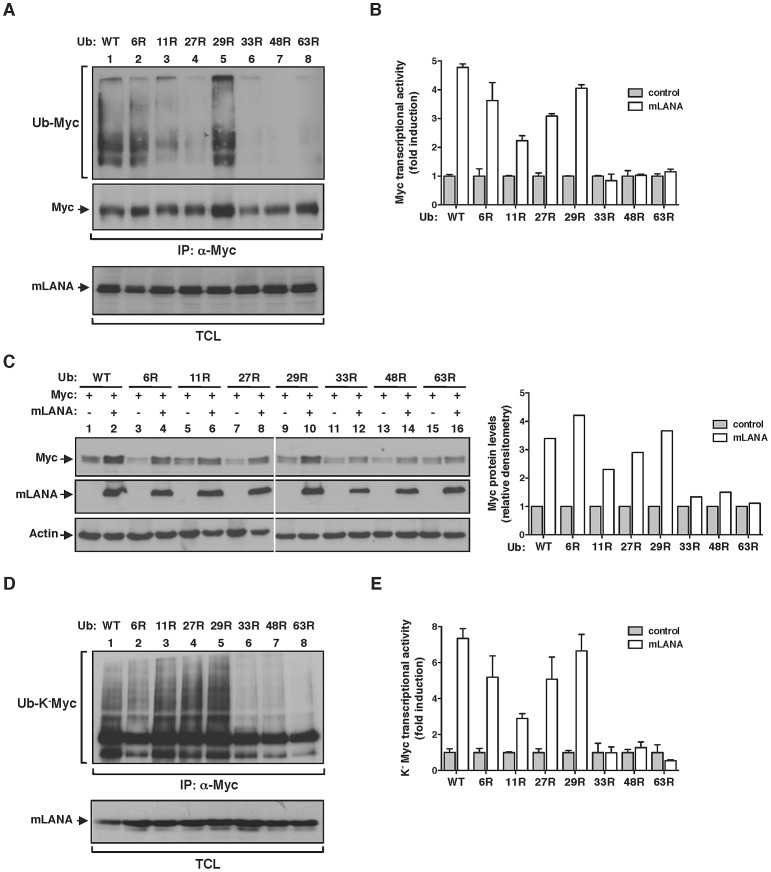
mLANA modulates Myc activity through the assembly of heterotypic poly-ubiqutin chains. (A) mLANA-mediated poly-ubiquitination of Myc is dependent on ubiquitin lysine residues (K) 33, 48 and 63. HeLa cells were transfected to express Myc, mLANA and wild type (WT) or mutant versions of ubiquitin, in which the indicated lysines had been replaced by arginines (R). TCL were subjected to denaturing immunoprecipitation using anti-Myc and analysed by immunoblotting with anti-ubiquitin. (B) mLANA stimulation of Myc transcriptional activity requires K33, K48, and K63 of ubiquitin. HeLa cells were transfected with a Myc (E-box) luciferase reporter vector, the indicated ubiquitin mutants, combined with (open bars) or without (filled bars) mLANA. Myc transcriptional activity was assayed as described in Figure 3C. Error bars represent SEM from triplicates from three independent transfection experiments. (C) mLANA-expressing cells exhibit increased Myc levels when K33, K48 and K63 residues of ubiquitin are preserved. HeLa cells were transfected to express the indicated combination of proteins (top) and Myc protein levels were analysed by immunoblotting. Right panel shows the densitometry analysis of Myc levels in mLANA transfected cells, expressed as fold induction relative to control transfected cells, normalised to Actin. (D) mLANA requires ubiquitin residues K33, K48 and K63 to mediate the poly-ubiquitination of the N-terminus of Myc. HeLa cells were transfected to express K^−^Myc, mLANA and the indicated ubiquitin mutants. Ubiquitination of K^−^Myc was assessed as described in A. (E) mLANA modulation of K^−^Myc transcriptional activity involves ubiquitin residues K33, K48 and K63. HeLa cells were transfected to express K^−^Myc, the indicated ubiquitin mutants, mLANA (open bars) or control transfected (grey bars). Myc transcriptional activity was assayed as described in Figure 3C. Error bars represent SEM from triplicates from three independent transfection experiments. −, without; +, with; α-, anti; IP, immunoprecipitation; TCL, total cellular lysates.

The addition of ubiquitin chains to a target protein can occur with different moieties that are emerging as determinants of biological outcome. These include, mono-ubiquitination and poly-ubiquitination. Ubiquin chains can be assembled using seven internal lysine residues (K) and thus define homotypic poly-ubiquitination (same K-linkage) or heterotypic poly-ubiquitination (mixed K-linkages) [26]. To investigate the type of lysine (K)-linkage involved in mLANA-mediated poly-ubiquitination of Myc we utilized of a series of ubiquitin mutants with every single lysine residue, of the possible seven, substituted by an arginine. By performing in vivo ubiquitination assays in mLANA expressing cells we observed that in the absence of K33, K48 or K63 residues the ability of mLANA to promote Myc poly-ubiquitination was suppressed (Figure 4A). Next, Myc transcription reporter assays were carried out in cells expressing the same ubiquitin mutants. Consistent with the poly-ubiquitination pattern, replacement of K33, K48 and K63 in ubiquitin rendered mLANA unable to positively modulate Myc transcriptional activity (Figure 4B). Measurement of Myc cellular levels in extracts from mock transfected control or mLANA-transfected cells co-expressing each ubiquitin mutant, revealed that in the presence of wild type ubiquitin, mLANA-expressing cells exhibit augmented Myc levels (Figure 4C, upper panel, compare lanes 1 and 2). The same result is observed when residues K6, K11, K27 and K29 of ubiquitin were substituted by arginines (Figure 4C, upper and lower panels). However, in good agreement with the previous data, substitution of K33, K48 and K63, diminished or abolished the ability of mLANA to promote the increase in Myc cellular levels (Figure 4C). Our observations demonstrate the involvement of different lysine-linkages in EC_5_S^mLANA^ Myc poly-ubiquitination. However, these data do not distinguish between homotypic poly-ubiquitination at different K residues in Myc from mixed K-linkage poly-ubiquitination. Hence, we next utilized K^−^Myc where ubiquitination is only possible at the first metionine. In vivo ubiquitination assays and transcription reporter assays with K^−^Myc, in combination with each of the K ubiquitin mutants, essentially recapitulated the above observed results with wild type Myc (Figure 4D and E). Hence, heterotypic poly-ubiquitination of the N-terminus of Myc is sufficient for mLANA modulatory activity. Collectively, these data show that EC_5_S^mLANA^ requires different K-linkages to ubiquitinate Myc. Moreover, they demonstrate a direct correlation between the ability of mLANA to mediate Myc poly-ubiquitination, to increase Myc cellular levels, and to promote its transcriptional activity, supporting that all three activities are directly linked.

### mLANA poly-ubiquitination on Myc is non-degradative

**Figure 5:**
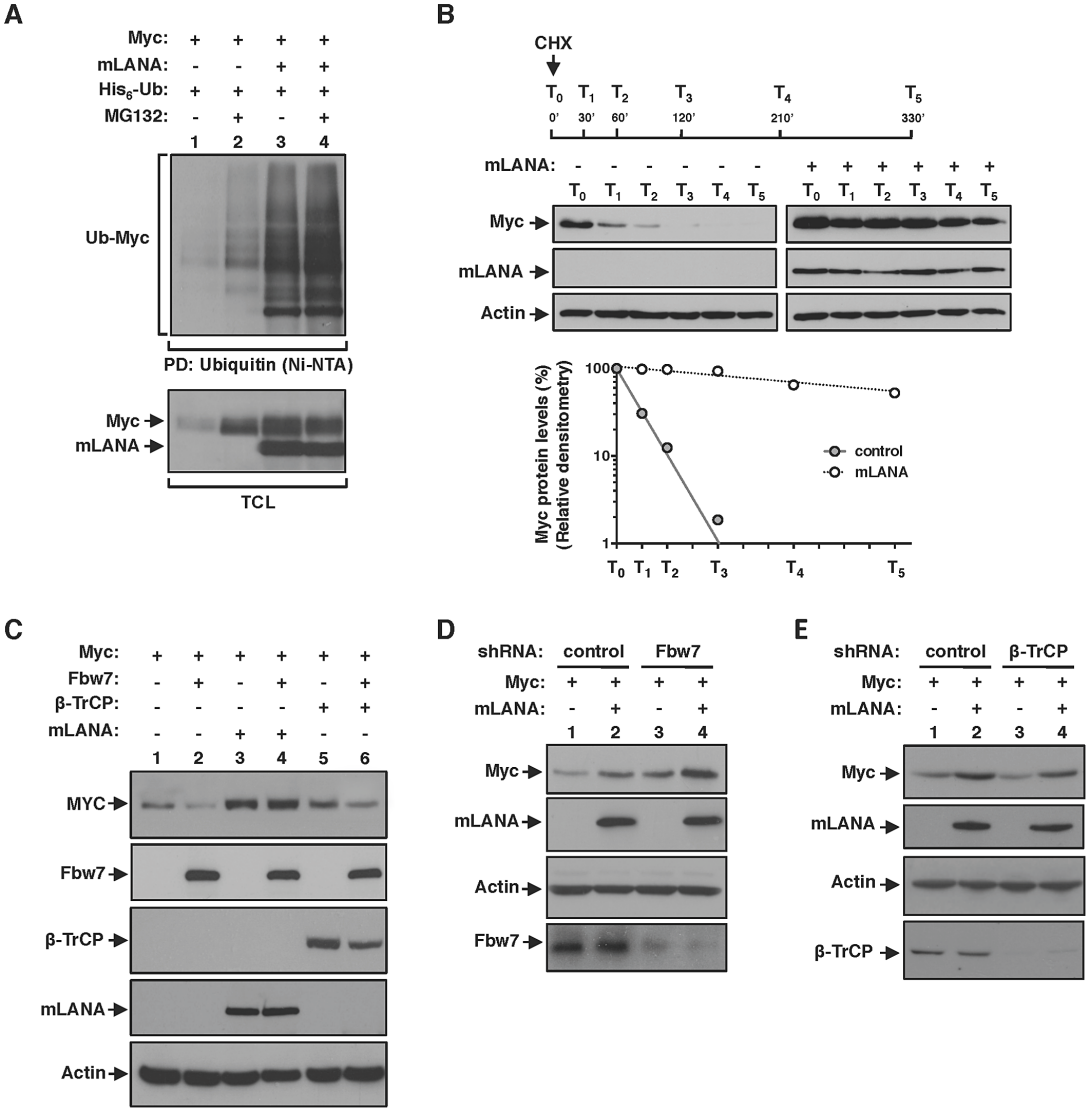
EC_5_S^mLANA^ mimics SCF^β-TrCP^ by antagonizing SCF^Fbw7^-mediated proteasomal turnover of Myc. (A) mLANA-mediated poly-ubiquitination of Myc is non-degradative. Cells transfected with the indicated plasmids were treated with MG132 (10 µM) for 8 h, or left untreated as depicted. Myc poly-ubiquitination was assayed as described in Figure 3F. (B) Myc half-life is increased in mLANA-expressing cells. HEK 293T cells were transfected with mLANA or control transfected. Cells were treated with cycloheximide (CHX, 100 µg/ml) for the times indicated (T_0_-T_5_). TCL were analysed by immunoblotting as indicated. Bottom panel shows the densitometry analysis of Myc protein levels present in control (filled circles) or mLANA transfected (open circles) cells, treated with CHX, normalised to Actin, assuming that Myc is 100% at time zero. (C) mLANA protects Myc from Fbw7-mediated turnover. HEK 293T cells were transfected with the indicated plasmids. Cellular lysates were obtained and Myc protein levels were assayed by immunoblotting. (D) Myc cellular levels in Fbw7 depleted cells are further increased by mLANA expression. HEK293T cells were transfected with the indicated shRNA vectors. After culture, Myc protein levels were analysed by immunoblotting. (E) mLANA is able to compensate the detrimental effect of β-TrCP depletion on Myc cellular levels. Cells were transfected with shRNA vectors and expressing plasmids, as depicted. Myc levels were analysed by immunoblotting. −, without; +, with; α-, anti; PD, pull-down; TCL, total cellular lysates.

Although classically associated with protein degradation, ubiquitination is now emerging as regulator of a wide variety of non proteolytic cellular signalling functions [26]. Therefore, and in agreement with a positive effect on Myc activity, we further confirmed that mLANA-mediated poly-ubiquitination of Myc was non-degradative. To that end, we performed an in vivo ubiquitination assay in the presence of the proteasome inhibitor MG132. In control-transfected cells, the presence of MG132 favoured the accumulation of Myc ubiquitinated species (Figure 5A, top panel, compare lanes 1 and 2), as well as the cellular levels of Myc protein (Figure 5A, bottom panel, compare lanes 1 and 2). In contrast, in cells expressing mLANA, inhibition of proteasomal degradation had a negligible influence on both Myc poly-ubiquitination and Myc protein levels (Figure 5A, compare lanes 3 and 4). The importance of these results is threefold. Firstly, they confirm that under physiological conditions Myc turnover is highly regulated by the proteasome. Secondly, they show that treatment with the proteasome inhibitor MG132 has no effect on mLANA-mediated poly-ubiquitination of Myc, thus mLANA in not directing Myc for proteasomal degradation. Thirdly, the increase in Myc levels in response to mLANA expression was not altered under conditions of proteasomal inhibition, suggesting that expression of mLANA is preventing the proteasomal degradation of Myc. To assess this hypothesis, we next compared the half-life of Myc in control and mLANA expressing cells. Cells were treated with the protein synthesis inhibitor cycloheximide (CHX), and Myc protein levels were analyzed by immunoblotting at different time-points post-treatment. Under these experimental conditions, expression of mLANA led to a pronounced raise in Myc stability with an increase of half-life from ≈20 minutes to over 4 hours (Figure 5B). Collectively these results demonstrate that mLANA expression has a positive effect on Myc cellular levels by preventing its proteasomal turnover, thus prolonging its half-life, which is associated with increased transcriptional activity.

### EC_5_S^mLANA^ mimics SCF^β-TrCP^ by antagonizing SCF^Fbw7^-mediated proteasomal turnover of Myc

Under physiological conditions Myc half-life is tightly regulated through poly-ubiquitination by two distinct Skp1/Cul1/F-box (SCF) E3 ubiquitin-ligases. That is, poly-ubiquitination of Myc by SCF^β-TrCP^ antagonizes SCF^Fbw7^-mediated proteasomal dependent turnover [6], [8]. Therefore, we hypothesised if mLANA could be modulating Myc stability by counteracting degradation by Fbw7 and mimicking the activity of β-TrCP. We overexpressed Fbw7 and analysed Myc cellular levels in the presence of co-expressed mLANA. As expected, overexpression of Fbw7 led to a decrease in Myc levels (Figure 5C, compare lanes 1 and 2). Overexpression of Fbw7 had no effect on Myc levels when mLANA was concomitantly expressed (Figure 5C, compare lanes 3 and 4). Notably, the antagonizing activity of mLANA towards Fbw7 was more pronounced when compared with that afforded by β-TrCP (Figure 5C, compare lanes 4 and 6). We further characterized the mLANA effect on Fbw7 and β-TrCP interplay by depletion of the expression of the two cellular E3 ubiquitin-ligases and analysis of Myc levels. When Fbw7 was depleted Myc levels increased further by the presence of mLANA (Figure 5D, compare lanes 3 and 4). In agreement, the turnover effect on Myc levels caused by depletion of β-TrCP was counteracted by concomitant expression of mLANA (Figure 5E, compare lanes 3 and 4). Together these results demonstrate that mLANA mimics SCF^β-TrCP^ by antagonizing SCF^Fbw7^-mediated proteasomal turnover of Myc.

### mLANA modulation of Myc activity is independent of the phosphorylation status of Myc

**Figure 6:**
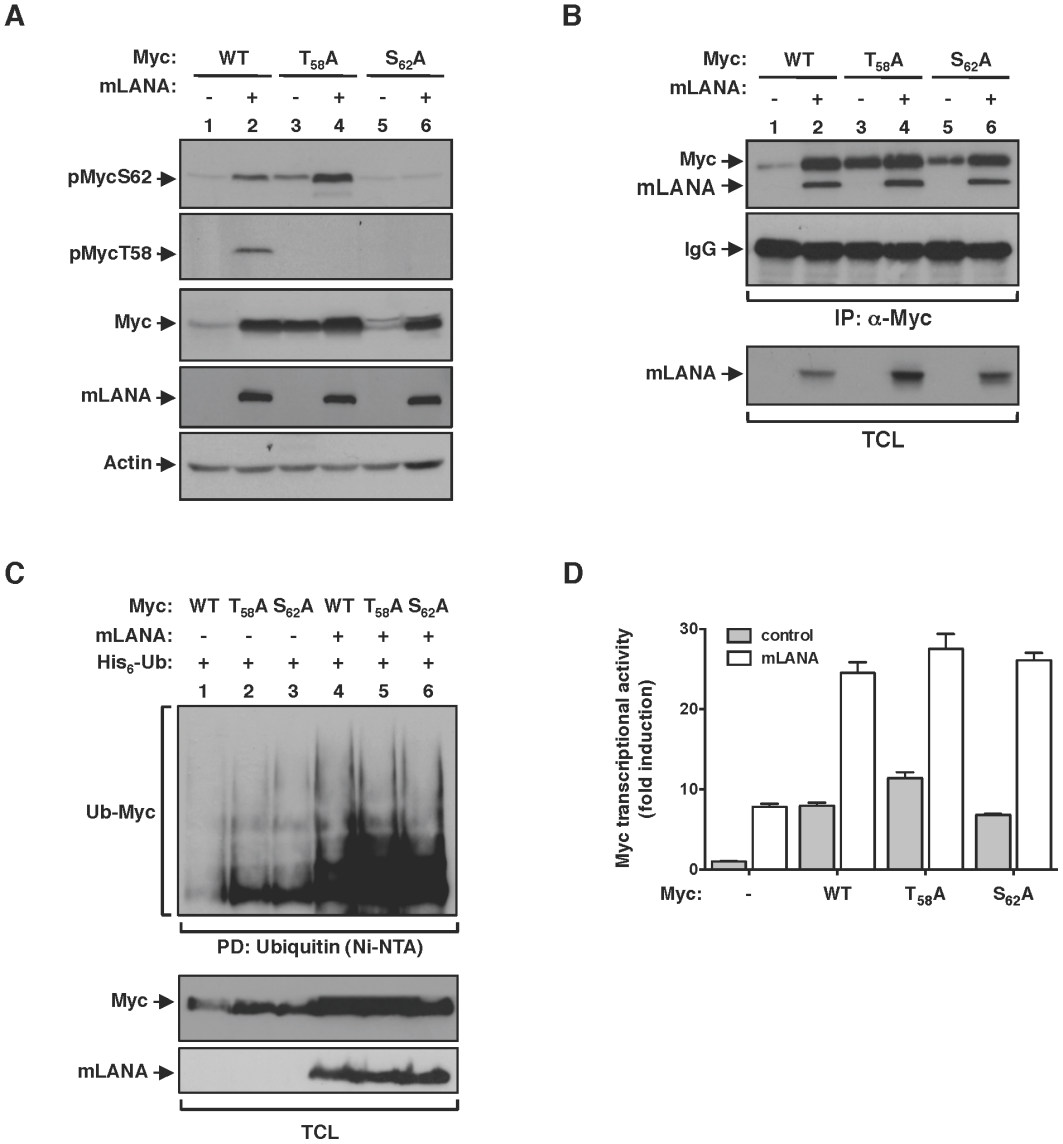
mLANA modulatory activity towards Myc is independent of Myc phosphorylation status. (A) HEK 293T cells were transfected to express wild type Myc protein, or specific T58 or S62 residues mutated to alanines, with or without mLANA, as indicated and subjected to immunoblotting analysis with phospho-specific antibodies directed towards Myc phosphorylated on S62 or T58. (B) In vivo interaction of Myc with mLANA is independent of its phosphorylation on T58 and S62 residues. Cellular extracts from HEK 293T cells transfected with the indicated plasmids were subjected to immunoprecipitation with anti-Myc and analysed by immunoblotting. (C & D) mLANA-mediated poly-ubiquitination and activation of Myc is independent of Myc phosphorylation on T58 and S62 residues. (C) HEK 293T cells were transfected with the indicated combinations of plasmids. Cellular lysates were obtained and subjected to Ni-NTA pull down as described in Figure 3F. (D) HeLa cells were transfected with a Myc (E-box) luciferase reporter vector combined with plasmids for Myc, Myc phospho-mutants, with (grey bars) or without (open bars) mLANA, as indicated and Myc transcriptional activity assayed as described in Figure 3C. Error bars represent SEM from triplicates from three independent transfection experiments. −, without; +, with; α-, anti; TCL, total cellular lysates.

Fbw7 control of Myc turnover is dependent on the interaction between the ubiquitin-ligase and its substrate. Fbw7 recognizes Myc when phosphorylated on threonine (T) 58 and catalyses its poly-ubiquitination resulting in Myc proteasomal degradation [6], [7]. Phosphorylation of Myc on T58 is sequentially preceded by phosphorylation on serine (S) 62, which activates and promotes Myc stability [5]. Thus T58 and S62 are key phospho-residues that regulate Myc activity at the protein level. Therefore, we set to investigate the influence of Myc phosphorylation on the modulatory activity of mLANA. Using phospho-specific antibodies we observed that under mLANA expression Myc is phosphorylated on both S62 and T58 (Figure 6A, lane 2, first and second panels, respectively). To analyse if sequential phosphorylation of Myc was intact on mLANA expressing cells we proceed to substitute S62 or T58 to alanines (A) on Myc and assess the phosphorylation status of those Myc mutants. Compatible with the model of sequential phosphorylation, in which phosphorylation of S62 precedes phosphorylation of T58, MycT_58_A was readily phosphorylated on S62, whereas MycS_62_A was not phosphorylated (Figure 6A, lanes 3–6, first and second panels, respectively). Remarkably, analysis of total Myc cellular levels revealed that, regardless of Myc phosphorylation on either S62 or T58, co-expression of mLANA led to increased Myc levels (Figure 6A, third panel). These data not only demonstrate that mLANA is not interfering with Myc phosphorylation, but also indicates that mLANA modulatory functions override cellular pathways that control Myc activity. Consistent with this hypothesis, mLANA is co-immunoprecipitated by both Myc mutants (Figure 6B), and it is able to mediate their poly-ubiquitination (Figure 6C). Transcriptional activities of MycT_58_A and MycS_62_A were also increased by expression of mLANA (Figure 6D), further supporting that mLANA targets Myc independently of cellular mechanisms of Myc regulation.

## Discussion

In this study, we describe the first example of a viral protein activating Myc transcriptional activity through increased protein stability by mimicking a physiological post-translational regulatory pathway. Modulation of Myc function was shown to be essential for the lymphotropic γ-herpesvirus MuHV-4 biology as infected cells exhibit increased expression of known Myc target-genes, and using a genetic approach, virus was found to amplify exclusively in intact Myc GC B-cells. The molecular mechanism involved heterotypic poly-ubiquitination of Myc mediated via the mLANA protein encoded by *ORF73*. This was reminiscent of a newly described pathway of Myc regulation through poly-ubiquitination. Popov et al. showed that the cellular E3 ubiquitin-ligase SCF^β-TrCP^ uses UbcH5 ubiquitin-conjugating enzyme to form heterotypic poly-ubiquitin chains on the N-terminus of Myc. Poly-ubiquitination of Myc by SCF^β-TrCP^ leads to Myc stabilization and was shown to antagonize SCF^Fbw7^-mediated proteasomal turnover of Myc [8]. Like SCF^β-TrCP^, EC_5_S^mLANA^ uses UbcH5 and antagonizes SCF^Fbw7^. Furthermore, as previously demonstrated for SCF^β-TrCP^, single substitutions of K33, K48 or K63 of ubiquitin reduced or eliminated the ability of EC_5_S^mLANA^ to poly-ubiquitinate, stabilize or increase the transcriptional activity of Myc. However, our results suggest that the molecular mechanism of mLANA modulation of Myc activity is not limited to Fbw7 antagonism. This is supported by the fact that mLANA protective effects on Myc stability, in conditions of Fbw7 over expression, are significantly more pronounced than observed for β-TrCP. Moreover, mLANA was able to increase the stability and activity of MycT58A, a Myc version that is not recognized by Fbw7. This contrasts with what has been reported for β-TrCP that albeit being able to poly-ubiquitinate MycT58A it did not impact on its turnover [8]. Thus, the ability of mLANA to increase Myc transcriptional activity through poly-ubiquitination, independently of the phosphorylation status of Myc on S58 and T62, indicates that this novel viral modulatory mechanism does not rely on post-translation cellular regulation.

Modulation of host B-cell biology is of vital importance for γ- herpesviruses, as they depend critically on the expansion of latently infected B-cell in GC reactions for host colonization. GC reactions exhibit two distinct morphological areas. These include the dark zone (DZ), where centroblasts are rapidly dividing, and the light zone (LZ), where B-cells exit the cell cycle to differentiate into plasma cells or memory B-cells or reenter the DZ for additional rounds of cell division. Recently two studies have established the importance of Myc for GC biology [13], [14]. These studies show that Myc expression is restricted to minute clusters of B-cells that initiate GCs and a small fraction of LZ B-cells. Furthermore, genetic interference with Myc expression or activity blocks GC formation and results in the collapse of mature GCs. Combined these studies demonstrate that expression of Myc is essential for the initiation and maintenance of GCs preceding B-cell proliferation in the DZ. It has been proposed that the lack of Myc expression in DZ B-cells, in conjunction with its short half-life, settles strict limits on the number of cell divisions afforded by centroblasts [27]. Therefore, by overriding the post-translational physiological control of Myc and significantly prolonging its half-life, mLANA favors an increase in the number of cell divisions during the expansion of MuHV-4 infected B-cells in the GC competitive niche. This modulation is likely to operate in infected B-cells at the initiation of a GC reaction and re-entry into the DZ. This interpretation is consistent with the observation that around 70% of MuHV-4 infected GC B-cells are rapidly dividing centroblasts versus approximately 20% infection in centrocytes. However, we show here that MuHV-4 is not able to latently expand in B-cells depleted of Myc expression. Hence, it is dependent on previous expression of Myc in B-cells. This is supported by our observation that MuHV-4 infected cells do not exhibit increased levels of Myc mRNA. Combined, our data is in good agreement with a post-translational mechanism of mLANA-mediated Myc stabilization in latently infected B-cells to increase their proliferative potential within GC reactions. This modulation is compatible with the selective expression of mLANA within GC B-cells [24] and its viral episomal maintenance properties [28].

By increasing the proliferative potential of latently infected GC B-cells through the modulation of Myc, γ-herpesviruses are effectively promoting host colonization. However, different γ-herpesviruses have evolved distinct mechanisms to accomplish Myc modulation. KSHV achieves this via LANA through a mechanism that involves targeting of Gsk-3 [17] whereas EBV appears to increase the transcription activity of Myc via interaction of EBNA3C with SCF^Skp2^ [18]. However, how vital is Myc modulation for γ-herpesvirus host colonization? We have previously reported that EC_5_S^mLANA^ mediates poly-ubiquitination-dependent proteosomal degradation of the NF-κB family member p65/RelA [25]. In that study we demonstrate that a recombinant MuHV-4 with a disrupted SOCS-box motif in mLANA loses the ability of the virus to expand in GC B-cells and persist in the mouse. Given that this recombinant lacks ubiquitin-ligase activity we cannot ascribe its phenotype to NF-κB or Myc modulatory effects since both activities depend on an intact SOCS-box motif. We show here that mLANA interacts with Myc through a motif independent of the SOCS-box and have shown before that this also applies to interaction with p65/RelA [25]. We have mapped both interactions to the N-terminal half of mLANA but have been unable to identify any discrete binding motif to both cellular targets (unpublished observations). Since structural modeling of the N-terminal half of mLANA predicts it to be unstructured we envisage that conformational rather than linear binding motifs may be required for interaction of mLANA with p65/RelA and Myc. Current mLANA structural studies in our laboratory are addressing this question. However, the property of mLANA to modulate both Myc and p56/RelA supports that maintenance of a proliferative GC reaction through Myc stabilization requires simultaneous inhibition of NF-κB signaling.

The molecular basis for EC_5_S^mLANA^-mediated poly-ubiquitination of Myc and p65/RelA resulting in opposed outcomes is not known. Effector proteins with ubiquitin binding domains (UBDs) trigger specific cellular responses by recognizing different types of ubiquitin topologies [26]. Hence, the decoration of Myc and p65/RelA with distinct K-linkages and lengths by EC_5_S^mLANA^ may determine distinct ubiquitin-mediated cellular functions. It is interesting to note that in this respect a parallel exists between EC_5_S^mLANA^ and SCF^β-TrCP^, as the latter also targets IκBα for proteasomal-mediated degradation [29] whereas it promotes Myc stabilization. The identification of UBD containing proteins that discriminate poly-ubiquitin chains topologies, which determine different biological outcomes is a field under intensive investigation. mLANA, therefore, provides as a good in vivo model for future studies.

Herein, we described a novel viral mechanism of stabilization of Myc through heterotypic poly-ubiquitination mediated by mLANA. The findings presented sustain the interpretation that increasing Myc stability is critical for the amplification of γ-herpesviruses in GC B-cells, thus persistence in the host. Therefore, this study provides a pathogenesis link between Myc and γ-herpesviruses associated lymphoproliferative disease.

## Materials and Methods

### Ethics statement

This study was carried out in strict accordance with the recommendations of the Portuguese official Veterinary Directorate (Portaria 1005/92). The Portuguese Experiments on Animal Act strictly comply with the European Guideline 86/609/EEC and follow the FELASA. Animal experiments were approved by the Portuguese official veterinary department for welfare licensing under the protocol number AEC_2010_017_PS_Rdt_General and the IMM Animal Ethics Committee.

### Reporter gene assays

For reporter gene assays, cells were transiently transfected with 500 ng of reporter vector, 1 µg of Myc and mLANA/mLANA–SOCS expression plasmids. In all transfections, a *Renilla* luciferase plasmid (10 ng) was used to normalise luciferase activity. Firefly and *Renilla* luciferase activities were assayed using Dual-Luciferase (Promega). Results are shown as fold induction relative to firefly luciferase activity measured in control-transfected cells.

### Cell cycle analysis

Cell cycle distribution profiles were analysed using Vybrant DyeCycle Violet Stain (Invitrogen) according to the manufacter's instructions. Briefly, 24 h post-tranfection with GFP, GFP-mLANA or GFP-mLANA-SOCS expressing plasmids, 1×10^6^ A20 cells were incubated with 1 µl of Vybrant DyeCycle in complete RPMI for 30 minutes, at 37°C. The percentage of cells in the various phases of cell cycle was determined using FlowJo software (Tree Star, Inc), implementing the Dean-Jett-Fox model. Three independent experiments were performed for each experimental condition and a representative experiment is shown.

### Immunoprecipitations

Cells were transiently transfected with plasmids encoding Myc or Myc phospho- mutants (2 µg), ubiquitin or ubiquitin with specific lysines mutated to arginines (4 µg), UbcH5 (2 µg) and/or mLANA or mLANA-SOCS (2 µg). Cells were disrupted in 10 mM Tris–HCl (pH 7.5), 150 mM NaCl, 1% Triton X-100, 1 mM NaF, 100 mM Na3VO4 and protease inhibitors (Complete; Roche). Supernatants were processed for immunoprecipitation as described [30]. Analysis of Myc or K^−^ Myc ubiquitination was performed under denaturing conditions (20 mM Tris–HCl (pH 7.5), 5 mM EDTA, 1% SDS, 10 mM dithiothreitol and Complete). Lysates were boiled for 10 minutes at 100°C, diluted 1/10 in lysis buffer and proceeded to immunoprecipitation.

### Myc ubiquitination in vivo

Levels of in vivo ubiquitinated Myc were determined by pull-down using Ni-NTA agarose beads. Cells were transfected with plasmids carrying His6-ubiquitin (4 µg), Myc (2 µg), and/or mLANA (2 µg). When indicated, cells were incubated for 8 hr in 10 µM MG132 (Calbiochem). Transfected cells were lysed in urea buffer (8M urea, 50 mM Tris-HCl (pH 7.5), 300 mM NaCl, 1% Triton X-100, 10 mM imidazole, 1 mM Na3VO4 and Complete), incubated for 2 hr at 4°C with Ni-NTA beads that were collected and washed with urea buffer. Proteins were eluted, denatured by boiling in Laemmli's buffer and analyzed by immunoblotting.

### Transcription analysis of Myc target genes

Total RNA from FACS-purified uninfected or infected GC B-cells, or transfected HEK 293T cells or A20 B cells was extracted with Trizol (Invitrogen). RNA (500 ng) was used for cDNA synthesis (DyNAmo, Finnzymes). qPCR was performed using DyNAmo Flash SYBR Green (Finnzymes). Primer sequences are available in Table S1. All reactions were run in duplicates. Amplification efficiencies and threshold cycle values were defined by the fractional cycle number at which fluorescence crosses the fixed threshold. Relative mRNA values, normalized to GAPDH, were calculated by the Pfaffl method [31].

### Laboratory animals

*Cγ1-Cre* mice were provided by Dr. Kurosaki (Japan), with the agreement of Dr. Rajewsky and Dr. Casola. *c-myc* floxed mice were a gift from Dr. Moreno de Alborán (Spain). *Cγ1-cre^KI/WT^*; *c-myc^fl/fl^* mice were generated by breeding heterozygous *Cγ1-cre* mice [22], with homozygous *c-myc^fl/fl^* mice [21]. *Cγ1-cre^WT/WT^*; *c-myc^fl/fl^* mice littermates were used as controls. C57BL/6 mice were obtained from Charles River Laboratories International Inc. Mice were bred and housed at IMM. All experimental protocols were performed in animals with 6–8 weeks of age.

### Immunization and virus assays

Immunizations were performed via intraperitoneal injection with 100 µg NP-CGG (Biosearch Technologies, Inc.) adsorbed to 3 mg of aluminium hydroxide (SERVA Electrophoresis GmbH), or 100 µg ovalbumin (OVA) grade V in CFA (Sigma). OVA immunized mice were challenged 7 days post-primary immunization with the same antigen/adjuvant combination. Inoculation of MuHV-4 was performed intranasally with 10^4^ p.f.u. in 20 µl of PBS under halothane. Frequencies of MuHV-4 genome-positive cells in GC B-cells were determined by limiting dilution combined with real-time PCR as previously described [32]. GC B cells were FACS purified from pools of five spleens using a BD FACSAria Flow Cytometer (BD Biosciences) and serially two-fold diluted. Eight replicates of each dilution were analysed by real time PCR (Rotor Gene 6000, Corbett Life Science). The primer/probe sets were specific for the MuHV-4 M9 gene (5′ primer: GCCACGGTGGCCCTCTA; 3′ primer: CAGGCCTCCCTCCCTTTG; probe: 6-FAM-CTTCTGTTGATCTTCC–MGB). Samples were subjected to a melting step of 95°C for 10 min followed by 40 cycles of 15 s at 95°C and 1 min at 60°C. Real-time PCR data was analyzed on the Rotor Gene 6000 software. The purity of sorted cells was always greater than 97%, as analyzed by flow cytometry.

### Plasmids, cell culture, DNA transfection and immunological reagents

Information provided in protocols S1.

## Supporting Information

Figure S1 **UbcH5 is required for mLANA poly-ubiquitination of Myc.** (A) mLANA immunoprecipitates exhibit E3 ubiquitin-ligase activity towards Myc, in vitro. Cell lysates from transiently transfected HEK 293T cells expressing mLANA or control transfected were subjected to immunoprecipitation with polyclonal anti-mLANA rabbit serum. After three washes in lysis buffer, immunoprecipitates were resuspended in reaction buffer (40 mM HEPES [pH 7.4], 60 mM potassium acetate, 1 mM EDTA, 2 mM DTT, 5 mM MgCl2, 10% glycerol). Myc was generated by transfection of 293T cells with a HA-tagged Myc expression plasmid, followed by immunoprecipitation with anti-HA antibodies after 48 hours of culture. After washing in lysis buffer, HA-Myc was eluted from beads using 0.5 mg/ml of HA peptide (Sigma). Reactions were supplemented with recombinant ubiquitin (2.5 µg) (Biomol International), E1 (50 ng), E2 (100 ng) (Calbiochem), GST-RelA (2.5 µg) and ATP regenerating buffer (Biomol International), when appropriate. Reactions were incubated for 1 hour at 30°C. Proteins were eluted in reduced Laemmli's buffer, resolved by SDS-PAGE and analyzed by immunoblotting with anti-ubiquitin antibody. (B) UbcH5 co-immunoprecipitates with mLANA dependent on mLANA SOCS-box motif. mLANA or mLANA-SOCS proteins were immunoprecipitated from total cellular lysates from HEK 293T cells transiently transfected with the expression plasmids (top). The presence of UbcH5 in the immunoprecipitates was analysed by immunoblotting. (C) mLANA-mediated activation of Myc is dependent on endogenous UbcH5 expression. HeLa cells were transfected with pSuper-puro vectors encoding control non-targeting (grey bars) or UbcH5 (open bars) directed shRNAs, along with the indicated expressing plasmids (bottom). Analysis of Myc transcriptional activity associated with each experimental condition was assessed as described in Figure 3C. Error bars represent the standard error of the mean from three independent experiments. −, without; +, with; α-, anti; IP, immunoprecipitation; TCL, total cellular lysates.
(TIFF)

Protocol S1 **Plasmids, cell culture, DNA transfection and immunological reagents.** Description of plasmids and immunological reagents utilized. Cell culture and DNA transfections procedures are described.
(DOCX)

Table S1 **Oligonucleotides used for transcriptional analysis of Myc target genes.** Primers were designed using qPrimerDepot database accessed at http://primerdepot.nci.nih.gov or http://mouseprimerdepot.nci.nih.gov for human or mouse genes, respectively. Primer sequences, NCBI Reference Sequence Accession.
(DOCX)
