# Supplementary figures and images for: Stabilization of Myc through Heterotypic Poly-Ubiquitination by mLANA Is Critical for γ-Herpesvirus Lymphoproliferation
Source: PLoS Pathog. 2013 Aug 8;9(8):e1003554. doi: 10.1371/journal.ppat.1003554 (PMC3738482; doi:10.1371/journal.ppat.1003554)

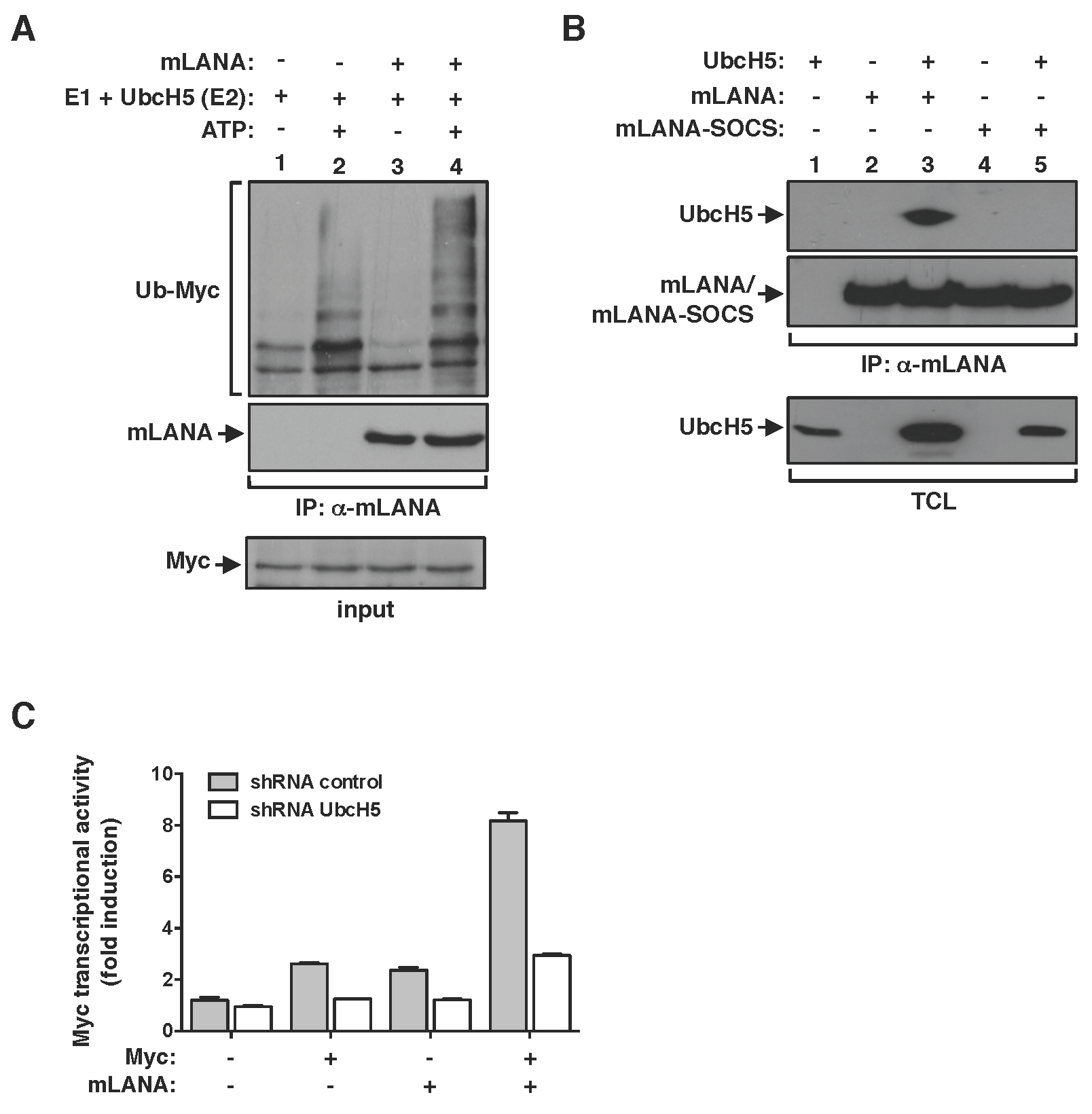

Supplement: Figure S1 — UbcH5 is required for mLANA poly-ubiquitination of Myc. (A) mLANA immunoprecipitates exhibit E3 ubiquitin-ligase activity towards Myc, in vitro. Cell lysates from transiently transfected HEK 293T cells expressing mLANA or control transfected were subjected to immunoprecipitation with polyclonal anti-mLANA rabbit serum. After three washes in lysis buffer, immunoprecipitates were resuspended in reaction buffer (40 mM HEPES [pH 7.4], 60 mM potassium acetate, 1 mM EDTA, 2 mM DTT, 5 mM MgCl2, 10% glycerol). Myc was generated by transfection of 293T cells with a HA-tagged Myc expression plasmid, followed by immunoprecipitation with anti-HA antibodies after 48 hours of culture. After washing in lysis buffer, HA-Myc was eluted from beads using 0.5 mg/ml of HA peptide (Sigma). Reactions were supplemented with recombinant ubiquitin (2.5 µg) (Biomol International), E1 (50 ng), E2 (100 ng) (Calbiochem), GST-RelA (2.5 µg) and ATP regenerating buffer (Biomol International), when appropriate. Reactions were incubated for 1 hour at 30°C. Proteins were eluted in reduced Laemmli's buffer, resolved by SDS-PAGE and analyzed by immunoblotting with anti-ubiquitin antibody. (B) UbcH5 co-immunoprecipitates with mLANA dependent on mLANA SOCS-box motif. mLANA or mLANA-SOCS proteins were immunoprecipitated from total cellular lysates from HEK 293T cells transiently transfected with the expression plasmids (top). The presence of UbcH5 in the immunoprecipitates was analysed by immunoblotting. (C) mLANA-mediated activation of Myc is dependent on endogenous UbcH5 expression. HeLa cells were transfected with pSuper-puro vectors encoding control non-targeting (grey bars) or UbcH5 (open bars) directed shRNAs, along with the indicated expressing plasmids (bottom). Analysis of Myc transcriptional activity associated with each experimental condition was assessed as described in Figure 3C. Error bars represent the standard error of the mean from three independent experiments. −, without; +, with; α-, an [file ppat.1003554.s001.tif]
