# Supplementary material for: Stabilization of Myc through Heterotypic Poly-Ubiquitination by mLANA Is Critical for γ-Herpesvirus Lymphoproliferation
Source: PLoS Pathog. 2013 Aug 8;9(8):e1003554. doi: 10.1371/journal.ppat.1003554 (PMC3738482; doi:10.1371/journal.ppat.1003554)
Supplement: Protocol S1 — Plasmids, cell culture, DNA transfection and immunological reagents. Description of plasmids and immunological reagents utilized. Cell culture and DNA transfections procedures are described. (DOCX) [file ppat.1003554.s002.docx]

**PROTOCOL S1**

**Plasmids**

mLANA and mLANA-SOCS expressing plasmids have been previously described (Rodrigues et al., 2009). mLANA and mLANA-SOCS in fusion with GFP were introduced into pCMV-Myc (Clontech). pMyc-TA-Luc vector, which contains six tandem copies of the E-box consensus sequence driving the expression of firefly luciferase, was purchased from Clontech. *Renilla* expression plasmid was a kind gift by Xosé R. Bustelo. Neil Perkins provided CMV-driven Myc encoding plasmid. Myc cDNA was amplified by PCR and subcloned to pCMV-HA (Clontech). T_58_-A and S_62_-A versions of Myc were generated by site-directed mutagenesis using QuikChange II XL Site-Directed Mutagenesis Kit (Agilent). Expression vectors encoding Fbw7 and β-TrCP were a gift from Markus Welcker and Bruce Clurman, respectively. Histidine-tagged ubiquitin plasmid was kindly offered by Dr D. Bohmann. HA-tagged ubiquitin expression vectors, shRNAs directed to target Fbw7, β-TrCP and UbcH5 were described before (Popov et al., 2010).

**Cell culture and DNA transfection**

HEK 293T and HeLa cells were cultured in Dulbecco’s modified Eagle’s medium containing 10% fetal calf serum, 2 mM glutamine, and 100 U/ml of penicillin and streptomycin. S11 and A20 cells were cultured in RPMI supplemented as above. HEK 293T and HeLa cells were transiently transfected with X-tremeGENE HP DNA Transfection Reagent (Roche Applied Science) according to the manufacturer’s instructions. A20 cells were transfected by electroporation (270 V, 500 μF), using a Bio-Rad gene pulser. In all transfections, empty vector was used to normalise the total amount of plasmid DNA. To measure Myc stability in vivo, 48 hours post-transient transfection, HeLa cells were treated with 100 μg/ml cycloheximide for the times indicated in Figure 6B.

**Immunological reagents**

mLANA mouse monoclonal antibody was raised against the C-terminal domain (aa 140-314) of the protein produced from *E. coli* as a GST-fusion protein. The antibodies against this region of mLANA were generated at the Monoclonal Antibody Core Facility, EMBL. Clones secreting mLANA specific antibodies were identified by ELISA and found to specifically recognize mLANA in transfected cells by western blotting and indirect immunofluorescence. All isolated clones recognized a region of mLANA comprising amino acids 140-168. Initial clones were further subcloned. A subclone (6A3) was expanded and identified as subclass IgG1 using an in-house ELISA protocol. Polyclonal mLANA antiserum was described before (Rodrigues et al., 2009). Anti-Myc (N-262), anti-Fbw7 (H-300), and anti-ubiquitin (P4D1) antibodies were purchased from Santa Cruz Biotechnology. Phospho-specific rabbit polyclonal antibodies to detect Myc phosphorylation on T_58_ (ab28842) and S_62_ (ab51156) were obtained from Abcam. β-TrCP (D13F10) and UbcH5 (A-615) antibodies were from Cell Signalling and BostonBiochem, respectively. Mouse monoclonal antibodies directed to epitope-tags HA (16B12), Myc (9E10) and Flag (M2) were from Covance, Clontech and Sigma, respectively. Actin was detected with an anti-actin rabbit polyclonal antibody (Sigma). Horseradish peroxidase conjugated secondary antibodies were from Amersham Biosciences. For FACS analysis of mouse splenocytes the following immunological reagents were used: anti-CD19 (1D3), anti-CD95 (Jo2), anti-IgM (R6-60.2), anti-IgD (11-26c-2a), anti-IgG1 (A85-1), anti-IgG2a/2b (R2-40), anti-CD86 (GL1), anti-CXCR4 (2B11) (BD Pharmigen) and anti-GL7 T and B Cell Activation Marker (GL-7) (eBioscience).
