# Supplementary material for: Stabilization of Myc through Heterotypic Poly-Ubiquitination by mLANA Is Critical for γ-Herpesvirus Lymphoproliferation
Source: PLoS Pathog. 2013 Aug 8;9(8):e1003554. doi: 10.1371/journal.ppat.1003554 (PMC3738482; doi:10.1371/journal.ppat.1003554)
Supplement: Table S1 — Oligonucleotides used for transcriptional analysis of Myc target genes. Primers were designed using qPrimerDepot database accessed at http://primerdepot.nci.nih.gov or http://mouseprimerdepot.nci.nih.gov for human or mouse genes, respectively. Primer sequences, NCBI Reference Sequence Accession. (DOCX) [file ppat.1003554.s003.docx]

**TABLE S1**

| **Gene** | **Acc. no** | **Forward primer 5’-3’** | **Reverse primer 5’-3’** | **Amplicon size** |
| --- | --- | --- | --- | --- |
| mouse *Ccnb1* | NM_172301 | GGCTTGGAGAGGGATTATCA | TGTGTGAACCAGAGGTGGAA | 114 |
| mouse *Ccnd1* | NM_007631 | GGGTGGGTTGGAAATGAAC | TCCTCTCCAAAATGCCAGAG | 110 |
| mouse *Ccnd2* | NM_009829 | CAGAGCTTCGATTTGCTCCT | TTCAGCAGGATGATGAAGTGA | 121 |
| mouse *Ccnd3* | NM_007632.2 | CGAGCCTCCTACTTCCAGTG | GGACAGGTAGCGATCCAGGT | 150 |
| mouse *Ccne1* | NM_007633 | GATTTTCCGAGGCTGAAATG | TGTGAAAAGCGAGGATAGCA | 139 |
| mouse *Cdk4* | NM_009870 | TTGTGCAGGTAGGAGTGCTG | TGCCAGAGATGGAGGAGTCT | 109 |
| mouse *Gapdh* | NM_008084 | TCAATGAAGGGGTCGTTGAT | CGTCCCGTAGACAAAATGGT | 125 |
| mouse *c-myc* | NM_010849 | ACGGAGTCGTAGTCGAGGTC | AGAGCTCCTCGAGCTGTTTG | 125 |
| mouse *Il10* | NM_010548 | GCTCTTACTGACTGGCATGAG | CGCAGCTCTAGGAGCATGTG | 105 |
| mouse *Batf* | NM_016767 | CTGGCAAACAGGACTCATCTG | GGGTGTCGGCTTTCTGTGTC | 108 |
| mouse *Mif* | NM_010798 | GCCAGAGGGGTTTCTGTCG | GTTCGTGCCGCTAAAAGTCA | 118 |
| mouse *Cd70* | NM_011617 | TGTAGCGGACTACTCAGTAAGC | TGGGGTCCTTCCGAGGAAC | 103 |
| human *Ccnb1* | NM_031966 | ACAGGTCTTCTTCTGCAGGG | GAACCTGAGCCAGAACCTGA | 124 |
| human *Ccnd1* | NM_053056 | GGCGGATTGGAAATGAACTT | TCCTCTCCAAAATGCCAGAG | 109 |
| human *Ccnd2* | NM_001759 | GCTTGGTCCAGTTCATCCTC | TGAGCTGCTGGCTAAGATCA | 147 |
| human *Ccne1* | NM_001238 | TCTTTGTCAGGTGTGGGGA | GAAATGGCCAAAATCGACAG | 110 |
| human *Cdk4* | NM_000075 | GGTCAAAGATTTTGCCCAAC | CCGAAGTTCTTCTGCAGTCC | 138 |
| human *Gapdh* | NM_002046 | TTAAAAGCAGCCCTGGTGAC | CTCTGCTCCTCCTGTTCGAC | 144 |
| human *c-myc* | NM_002467 | CACCGAGTCGTAGTCGAGGT | GCTGCTTAGACGCTGGATTT | 114 |
